# The Transcriptome of Human Epicardial, Mediastinal and Subcutaneous Adipose Tissues in Men with Coronary Artery Disease

**DOI:** 10.1371/journal.pone.0019908

**Published:** 2011-05-16

**Authors:** Sandra Guauque-Olarte, Nathalie Gaudreault, Marie-Ève Piché, Dominique Fournier, Pascale Mauriège, Patrick Mathieu, Yohan Bossé

**Affiliations:** 1 Centre de Recherche Institut Universitaire de Cardiologie et de Pneumologie de Québec, Laval University, Quebec, Canada; 2 Division of Kinesiology, Department of Social and Preventive Medicine, Faculty of Medicine, Laval University, Quebec, Canada; 3 Department of Molecular Medicine, Laval University, Quebec, Canada; Universitätsklinikum Schleswig-Holstein - Campus Luebeck, Germany

## Abstract

**Background:**

The biological functions of epicardial adipose tissue (EAT) remain largely unknown. However, the proximity of EAT to the coronary arteries suggests a role in the pathogenesis of coronary artery disease (CAD). The objectives of this study were to identify genes differentially regulated among three adipose tissues, namely EAT, mediastinal (MAT) and subcutaneous (SAT) and to study their possible relationships with the development of cardiovascular diseases.

**Methods and Results:**

Samples were collected from subjects undergoing coronary artery bypass grafting surgeries. Gene expression was evaluated in the three adipose depots of six men using the Illumina® HumanWG-6 v3.0 expression BeadChips. Twenty-three and 73 genes were differentially up-regulated in EAT compared to MAT and SAT, respectively. Ninety-four genes were down-regulated in EAT compared to SAT. However, none were significantly down-regulated in EAT compared to MAT. More specifically, the expression of the adenosine A1 receptor (ADORA1), involved in myocardial ischemia, was significantly up-regulated in EAT. Levels of the prostaglandin D2 synthase (PTGDS) gene, recently associated with the progression of atherosclerosis, were significantly different in the three pairwise comparisons (EAT>MAT>SAT). The results of ADORA1 and PTGDS were confirmed by quantitative real-time PCR in 25 independent subjects.

**Conclusions:**

Overall, the transcriptional profiles of EAT and MAT were similar compared to the SAT. Despite this similarity, two genes involved in cardiovascular diseases, ADORA1 and PTGDS, were differentially up-regulated in EAT. These results provide insights about the biology of EAT and its potential implication in CAD.

## Introduction

Increased visceral adipose tissue has been associated with the development of cardiovascular diseases [1], [2], [3]. EAT is the visceral fat depot located on the surface of the heart [4] especially around the epicardial coronary vessels with extension into the myocardium [5], [6], [7]. As shown by clinical imaging and histological studies, EAT increases with obesity [5], [8] and correlates with intra-abdominal fat mass [9]. Adipose tissue is now considered as a metabolically active organ which produces hormones and pro-inflammatory factors, contributing to the adverse cardiovascular consequences of obesity [1], [5].

The function of EAT is not completely understood [7], possible roles have been suggested such as lipid storage and endocrine organ [4], [5]. It is also an active inflammatory tissue known to secret cytokines and chemokines [10], [11], which are key factors involved in atherosclerosis [12]. EAT correlates with the presence of CAD [5] and is likely to be implicated in its pathogenesis. Previous studies in humans and rabbits demonstrated that segments of an artery surrounded by EAT develop atherosclerosis at a faster rate compared to the intra-myocardial segments of the same artery [1], [13]. Therefore, EAT may play a role especially in the severity of the pathology [14]. EAT has the potential to be both beneficial and damaging for the heart function. It is known to collect the excess of circulating fatty acids, which induce cardiotoxicity [5], and interfere with the generation and propagation of the contractile cycle of the heart, causing ventricular arrhythmias and alterations in repolarization [14]. In addition, due to its high rate of lipolysis, EAT may serve as a source of free fatty acids (FFA) to supply myocardial energy demand in times of need, especially under ischemic conditions [5], [14]. In contrast, EAT thickness was significantly correlated with the severity of CAD [7] and reflects carotid artery stiffness, an early manifestation of atherosclerosis [15]. Iacobellis et al. [9] have recently proposed that echocardiographic EAT thickness measurement might be useful for cardiometabolic risk stratification.

A number of properties differentiate EAT from other fat depots, specifically its smaller adipocyte size, low mRNA expression for several key enzymes involved in lipogenesis (lipoprotein lipase, stearoyl-CoA desaturase and acetyl-CoA carboxylase-α) [5], [14], slow regression during weight loss [16], and different fatty acid composition [17], among others [5]. These differences suggest that EAT presents specific gene regulation patterns. Only two studies have compared genome-wide transcriptional profiles of EAT with SAT from the same individuals [10], [18]. Moreover, no studies have compared the genome-wide mRNA profile of EAT with MAT and SAT from the same individuals.

In the present study, we compared the transcriptional profile of EAT with that of MAT and SAT from the same individuals by using whole-genome gene expression microarrays. We found that EAT and MAT share highly similar mRNA profiles. However, few genes involved in cardiovascular diseases were up-regulated in EAT compared to MAT. The expression of the genes ADORA1, involved in myocardial ischemia and dilated cardiomyopathy [19], and PTGDS, recently associated with the progression of atherosclerosis [20], [21] were significantly up-regulated in EAT.

## Results

Table 1 shows the clinical characteristics of the subjects. BMI ranged between 24.6 and 30.5 kg/m^2^ for subjects used in the microarray experiment (n = 6) and between 22.8 and 35.6 kg/m^2^ for subjects used in the qPCR experiment (n = 25). No significant differences were observed in clinical characteristics between patients used in the microarray and qPCR experiments.

**Table 1:** Clinical characteristics of the study participants.

	Microarrays subjectsn = 6	qPCR subjectsn = 25	pvalue
Age (years)	65.5±12.6	60.2±9.0	0.307
BMI (kg/m^2^)	26.2±2.2	27.9±3.4	0.255
Waist circumference (cm)	104.1±10.2	101.4±9.2	0.593
Hypertension	83.3% (5)	56% (14)	0.363
Dyslipidemias	100% (6)	100% (25)	1.000
Type 2 Diabetes	16.6% (1)	32% (8)	0.642
ACEI or ARA	83.3% (5)	64% (16)	0.634
HMG-CoA reductase inhibitors	100% (6)	92% (23)	1.000

ACEI: angiotensin-converting enzyme inhibitors; ARA: angiotensin receptor antagonist; BMI: body mass index; HMG-CoA reductase inhibitors: hydroxy-methylglutaryl-coenzyme A reductase inhibitors.

Continuous variables are expressed as mean ± SD and tested using t-tests. Dichotomous variables are expressed as percentage (n) and tested using Fisher's exact tests.

### Microarrays

The intrapair correlation coefficients for each tissue were obtained from normalized expression data and ranged from 0.96 and 0.99 for EAT and SAT; and from 0.94 and 0.98 for MAT (Figure S1).

Table 2 shows the number of genes that were differentially up- and down-regulated in each pairwise comparison. A total of 28 probes were significantly differentially up- or down-regulated between EAT and MAT, 199 between EAT and SAT, and 237 between MAT and SAT. The Tables S1, S2, and S3 list all the differentially up- and down-regulated probes for the comparisons EAT vs MAT, EAT vs SAT, and MAT vs SAT, respectively. These probes correspond to 306 unique genes among the three pairwise comparisons. Briefly, 23 and 73 genes were differentially up-regulated in EAT compared with MAT and SAT, respectively. 94 genes were differentially down-regulated in EAT compared to SAT, whereas no gene was down-regulated in EAT compared to MAT. Compared to SAT, 116 genes were differentially up-regulated and 74 genes differentially down-regulated in MAT.

**Table 2:** Number of genes up- and down-regulated in each pairwise comparison.

	EAT vs MAT		EAT vs SAT		MAT vs SAT	
	Probes	Genes	Probes	Genes	Probes	Genes
Up	28	23	85	73	143	116
Down	0	0	114	94	94	74
**Total**	**28**	**23**	**199**	**167**	**237**	**190**

ADORA1 was the most significant differentially up-regulated gene in EAT compared to MAT. The corresponding probe (ILMN_1747227) interrogated the 3′UTR region of the ADORA1 transcript (NM_000674.1) and provided a fold change of 2.9 and a FDR of 0%. However two other probes testing a different transcript (NM_000674.2) of the same gene were not significant. PTGDS was the only gene significantly different in the three comparisons with the highest expression in EAT followed by MAT and SAT. The overlap of genes differentially expressed between the 3 comparisons is shown in a Venn diagram (Figure S2).

**Figure 1:**
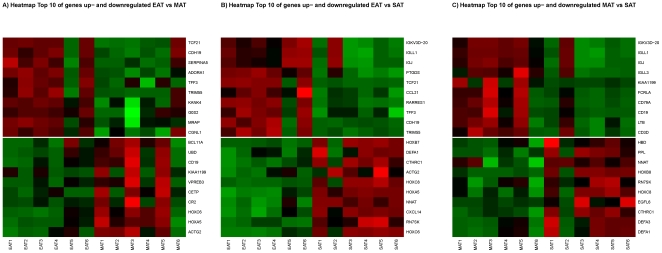
Heat map of the top 10 genes differentially up- and down-regulated for the three pairwise comparisons. The samples and genes are illustrated in columns and rows, respectively. Red and green represent high and low expression, respectively. The full names and biological processes related with these genes are provided in Tables S4, S5, and S6.

Figure 1 shows a heat map of the top 10 genes differentially up- and down-regulated for the three pairwise comparisons. Full annotations and main biological processes related with these genes can be found in Tables S4, S5, and S6. Briefly, 5 and 9 genes in the top ten genes were involved in inflammatory and immune response when comparing EAT and MAT with SAT, respectively. Furthermore, among the 116 genes differentially up-regulated in MAT vs SAT, 38 genes (33%) are involved in inflammatory response, such as chemokines and immunoglobulines. Compared to SAT, 23% (17 of 73) of the differentially up-regulated genes in EAT are involved in inflammatory response.

Figure S3 shows a heat map of the expression levels of genes that are specific for various lineages of white blood cells in the three adipose tissue compartments. Genes specific for B cells (CD79A and CD79B) were differentially up-regulated in MAT vs SAT. CD247 that is specific for T cells was also differentially up-regulated in MAT vs SAT. However the other two genes specific for T cells (CD3EAP and CD3G) were not different in this comparison. Specific genes for myeloid cells, monocytes and megakaryocytes were not different in any of the three comparisons.

### Mapping of genes to biological pathways

Transcripts deemed significant were analyzed using IPA, PANTHER, and ToppGene in order to identify molecular pathways and functional assignments that differ between adipose tissue compartments. In the comparison EAT vs MAT, three groups of *Toxicity functions* were significant, namely cardiotoxicity, hepatotoxicity, and nephrotocicity. Five genes significantly up-regulated were involved in cardiotoxicity: acyl-CoA synthetase long-chain family member 1 (ACSL1), adenosine A1 receptor (ADORA1), collagen type VI alpha 6 (COL6A6), cysteine-rich secretory protein LCCL domain containing 2 (CRISPLD2), and SRY (sex determining region Y)-box 9 (SOX9). Furthermore, ACSL1 was also involved in hepatotoxicity and PTGDS in nephrotocixity.

**Figure 2:**
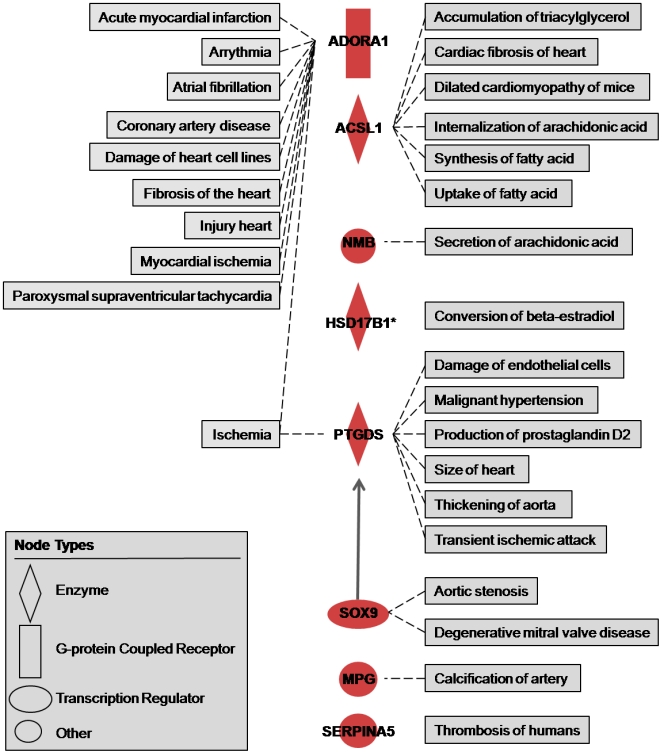
Cardiovascular functions linked to up-regulated genes in EAT compared to MAT. ACSL1, Acyl-CoA synthetase long-chain family member 1; ADORA1, adenosine A1 receptor; HSD17B1, hydroxysteroid (17-beta) dehydrogenase 1; MGP, matrix Gla protein; NMB, neuromedin B; PTGDS, prostaglandin D2 synthase; SERPINA5, serpin peptidase inhibitor -clade A (alpha-1 antiproteinase, antitrypsin)- member 5; and SOX9, SRY (sex determining region Y)-box 9. *Interaction with itself. →Acts on.

**Figure 3:**
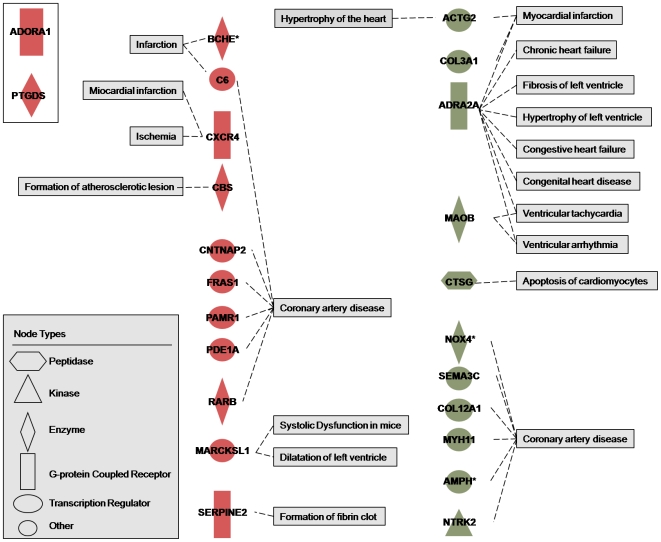
Cardiovascular functions linked to up- (red) and down-regulated (green) genes in EAT compared to SAT. ACTG2, actin, gamma 2, smooth muscle, enteric; ADRA2A, adrenergic, alpha-2A-, receptor; AMPH amphiphysin; BCHE, butyrylcholinesterase; C6, complement component 6; CBS, cystathionine-beta-synthase; CNTNAP2, contactin associated protein-like 2; COL3A1, collagen, type III, alpha 1; COL12A1, collagen, type XII, alpha 1; CTSG, cathepsin G; CXCR4, chemokine (C-X-C motif) receptor 4; FRAS1, Fraser syndrome 1; MAOB, monoamine oxidase B; MARCKSL1, MARCKS-like 1; MYH11, myosin, heavy chain 11, smooth muscle; NOX4, NADPH oxidase 4; NTRK2, neurotrophic tyrosine kinase, receptor, type 2; PAMR1, peptidase domain containing associated with muscle regeneration 1; PDE1A, phosphodiesterase 1A calmodulin-dependent; RARB, retinoic acid receptor beta; SEMA3C, sema domain, immunoglobulin domain (Ig), short basic domain, secreted, (semaphorin) 3C; and SERPINE2, serpin peptidase inhibitor clade E (nexin, plasminogen activator inhibitor type 1), member 2. *Interaction with itself.

The 22 significant genes in the comparison EAT and MAT were analyzed using the *Function and disease* tool in IPA. As a result, 8 genes significantly up-regulated in EAT vs MAT were related to cardiovascular functions (Figure 2). Interestingly ADORA1 was the most significant gene differentially expressed between EAT and MAT, but also the gene more highly linked to cardiovascular diseases in IPA. The same analysis was performed for the significantly up-regulated genes in the comparison EAT vs SAT. In addition to ADORA1 and PTGDS, 11 genes were related to CAD functions. Similarly, 11 significant down-regulated genes were related with cardiovascular functions in the same comparison (Figure 3).

In PANTHER, the only significant Biological process in the comparison EAT vs MAT was Heart development. Using ToppGene the single molecular function that was significant in this same comparison was Neuropeptide receptor binding. In the comparison EAT vs SAT several molecular functions and biological processes were called significant including the chemokine activity function, cardiovascular system development and inflammatory response processes. For the comparison MAT vs SAT most of the significant molecular functions, pathways and biological processes are related with immune and inflammatory response.

### qPCR

**Figure 4:**
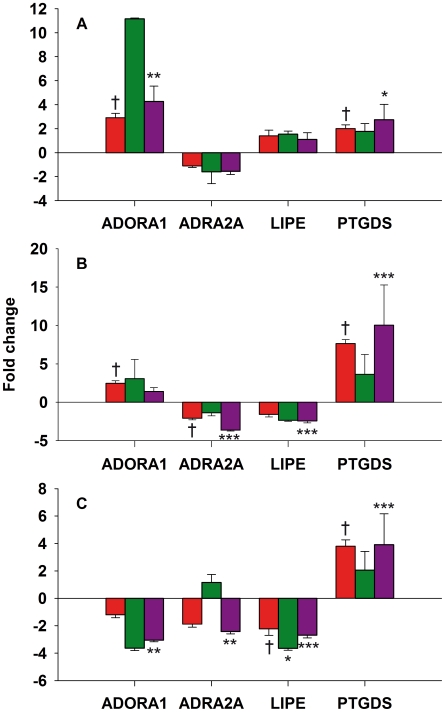
Comparison of fold changes for ADORA1, ADRA2A, LIPE, and PTGDS. EAT vs MAT (A), EAT vs SAT (B) and MAT vs SAT (C). The red bars represent the fold changes obtained by microarrays. The green and violet bars represent the fold changes obtained by qPCR for the microarrays and qPCR subjects, respectively. †Significant in the microarray experiment. *p<0.05, **p<0.001, and ***p<0.0001.

Validation by qPCR was performed in 25 independent subjects. Four genes were selected for this validation including ADORA1, the most significantly gene in the comparison EAT vs MAT (q-value 0.0); ADRA2A, differentially down-regulated in EAT vs SAT; LIPE, differentially down-regulated in MAT vs SAT; and PTGDS, the only gene differentially up-regulated in both EAT and MAT compared to SAT which is also associated with several cardiovascular functions. The differential regulations observed in the microarray experiment were confirmed for ADORA1 (EAT vs MAT), ADRA2A, LIPE and PTGDS (Figure 4). Although the comparison of ADORA1 between EAT and SAT was not significant by qPCR, the fold changes obtained by microarrays and qPCR followed the same trend (up-regulated, Figure 4 middle panel).

Using the 6 microarray subjects (Figure 4, green bars), only the gene LIPE was significantly differentially regulated comparing MAT with SAT. However, except for ADRA2A in the comparison MAT vs SAT, all the genes followed the same direction (up- or down-regulated). It should be noted that ADRA2A was not significant for this comparison in the microarray experiment.

There was no significant difference in the expression of the 4 genes tested by qPCR between patients with and without type 2 diabetes. Furthermore, no significant correlation was observed between the expression of these four genes and BMI.

## Discussion

Cardiovascular disease (CVD) is a leading global cause of death. Previous studies found a significant relation between EAT volume and the presence of CAD [22], but the role of EAT in CAD and other CVDs is still unclear. To expand our knowledge about the function of EAT and its relation with CVDs, we performed a microarray experiment to compare the gene expression profile of EAT, MAT, and SAT from subjects with CAD.

A total of 306 genes were significantly differentially regulated among three adipose tissues comparisons. Several genes involved in inflammatory and immune responses were identified among the top differentially up-regulated genes comparing EAT and MAT with SAT. Likewise, Mazurek et al. [10] found that EAT exhibited significantly higher expression levels of chemokines, inflammatory cytokines, and immune response genes compared to SAT, independently of obesity and diabetes. In addition, other studies [10], [23] showed that when compared to SAT the omental fat, incidentally a visceral fat with similar embryologic origin to EAT, express higher level of IL-6 and other cytokines. Another study [24] has correlated the thickness of EAT with the plasma levels of chemokine (C-C motif) ligand 2 (CCL2) and soluble IL-6 receptor/IL-6 in obese subjects. A previous microarray experiment identified the pro-inflammatory enzyme phospholipase A2 group IIA (PLA2G2A) as the top gene encoding for a secreted protein that was differentially up-regulated in EAT compared to SAT in both patients with or without CAD [18]. The single probe that interrogated this gene in our study was also differentially up-regulated in EAT compared to SAT (fold change = 1.20), but was not significant. Another study suggested that the expression of adiponectin (ADIPOQ) was lowered in EAT compared to SAT [25]. Again our results were in the same direction (fold change = 0.84 comparing EAT vs SAT), but not significant. A larger sample size may be required to confirm the differential expression of PLA2G2A and ADIPOQ between EAT and SAT.

We observed highly similar mRNA expression patterns between EAT and MAT. Only 23 genes were differentially expressed between these tissues. Mahabadi et al. [26] showed that the amount of pericardial and intra-abdominal fat, but not MAT, were significantly associated with CVD. Hence, the location of EAT over the myocardium and its direct contact with the coronary arteries is supporting the idea that local and paracrine effects of secreted molecules by EAT have the potential to influence the development of CAD and/or myocardial dysfunction.

We validated four genes by qPCR. These genes were selected because they were among the top differentially regulated genes, but also based on their importance in CAD and the biology of the adipose tissue. ADORA1 was the most significant differentially up-regulated gene in the microarrays experiment comparing EAT with MAT. It is a G protein coupled-receptor (GPCR), which couples to Gi to decrease the secondary messenger cAMP. ADORA1 agonists have potential therapeutic uses to treat CVD [27]. The activation of ADORA1 has an important role in protecting the myocardium from ischemic damage. Under ischemic conditions, the concentration of extracellular endogenous adenosine increases as a protective mediator. Up-regulation of ADORA1 in the heart of transgenic mice provides additional cardioprotection to that offered by adenosine [28], [29]. In these mice there was a decrease in cell necrosis, improvement of myocardial energy, and recovery after ischemia [28], [29]. Exogenous ADORA1 and ADORA3 agonists reduced cardiac infarct size and improved functional recovery in isolated heart models [30]. Myocardial injuries after ischemia-reperfusion induce apoptosis, and ADORA1 overexpression attenuated this apoptotic response [28]. Taken together, these studies confirm the importance of ADORA1 in the pathobiology of heart diseases, but do not explain how the differential up-regulation of this receptor in adjacent adipose tissues may affect myocardial or vascular function. In this regard, binding of adenosine to ADORA1 is well recognized to inhibit lipolysis, and omental fat cells are less responsive to this nucleoside than subcutaneous ones [31] because of a lower ADORA1 density in the former adipocytes [32]. Accordingly it is tempting to speculate that differentially up-regulation of ADORA1 is a negative feedback mechanism to attenuate the higher FFA release observed in EAT [5].

ADRA2A belongs to the adrenergic receptors family of GPCRs which mediates the effect of the endogenous catecholamines. It plays a central role in the regulation of systemic sympathetic activity and hence cardiovascular responses such as heart rate and blood pressure [33]. ADRA2A is important in the regulation of both cardiovascular and endocrine systems [34]. Targeted disruption of this gene causes hypertension, tachycardia and impaired baroreceptor reflexes in mice [35], [36]; and its overexpression in pancreatic islets contributes to type 2 diabetes [37]. The DraI allele was previously associated with increased sympathetic activity, thereby predisposing to hypertension [35]. In our study, ADRA2A was significantly differentially down-regulated in EAT and MAT compared to SAT. Similarly, Vohl et al. [23] observed that ADRA2A is differentially down-regulated in omental compared to SAT. Thus, the lower expression of ADRA2A in EAT and omental adipose tissues may suggest a lower sensitivity to antilipolytic signals as already confirmed by Mauriège et al. [38], [39] and Vikman et al. [40]. It is known that the rate of lipolysis in EAT is increased compared to other fat depots such as popliteal or perirenal [5], which might be in part mediated by a low expression of ADRA2A.

LIPE is an enzyme with lipolytic properties and catalyzes the rate-limiting step of lipolysis by hydrolyzing the stored triacylglycerols and diacylglycerols into fatty acids and glycerol [41]. In our study, LIPE was significantly differentially down-regulated in EAT and MAT compared to SAT. This finding is concordant with the higher maximum lipolytic capacity, LIPE activity and mRNA expression in subcutaneous abdominal compared to omental fat cells [42]. Yet, visceral fat cells are known to have higher lipolytic activity compared to subcutaneous adipocytes. This situation may be partly explained by increased insulin action and alpha 2-adrenergic receptor mediated antilipolysis observed in subcutaneous fat depots [38], [40], [43]. In contrast, Fain et al. [44] found that EAT expressed higher levels of LIPE compared to SAT in a mixed population of men and women. However, our findings also support those of Vohl et al. [23] who found that LIPE was differentially down-regulated in visceral fat compared to SAT in men. Other studies have found no difference in the expression of LIPE between visceral and subcutaneous fat [45], [46]. Differences in adipose tissue gene expression between men and women may account for such disparities [47], [48]. Cellular heterogeneity may also be considered as Montague et al. [45] studied isolated adipocytes (being separated from the stromal-vascular cells of the adipose tissue), while the whole adipose tissue was homogenized before RNA extraction in our study and the one of Vohl et al. [23]. Since LIPE is well known to be differentially up-regulated in adipocytes compared to preadipocytes [49], and as it is also expressed in macrophages [50], cell heterogeneity in whole adipose tissue biopsies might explain the differences between studies.

PTGDS is an enzyme involved in the synthesis of prostaglandin D2 (PGD2) [51]. PGD2 is a potent anticoagulant and vasodilatator [20]. PTGDS overexpression is thought to be an adaptative mechanism to prevent cardiovascular injuries because of its anti-inflammatory properties [51]. The PTGDS bioproducts PGD2 and 15d-PGJ2 lead to a reversion of the inflammatory state and plaque stabilization in human atherosclerosis [52]. PTGDS knockout mice developed atherosclerosis when compared with controls [53]. Its expression may be increased to protect against platelet aggregation in atherosclerotic blood vessels [54] and to inhibit the growth phenotype of vascular smooth muscle cells [54]. Moreover, PTGDS is considered a suitable biomarker of CAD as its levels in serum increase in subjects with stable CAD and with the severity of the disease [20]. In the present study, PTGDS was significantly differentially up-regulated in EAT compared to MAT and SAT. This finding corroborates the study by Fain et al. [44], which showed higher levels of this transcript in EAT compared to substernal and subcutaneous fat in a group of men and women undergoing CABG [44]. The fact that the coronary arteries and their main epicardial branches are embedded in EAT [5] suggests that part of the higher PTGDS levels observed in atherosclerotic plaques of coronary arteries, compared to the internal mammary or the carotid arteries [20], may come from EAT. In our study, two other genes involved in the arachidonic acid metabolism were also differentially up-regulated in EAT, including ACSL1, the first enzyme in the incorporation of arachidonic acid into phospholipids, and NMB, that stimulates arachidonic acid release. This suggests the utilization of arachidonic acid and the production of pro-inflammatory mediators by EAT. Whether this differentially up-regulation of genes involved in the arachidonic metabolism is a cause or a consequence of CAD remains to be elucidated.

This study has some limitations. First, it was limited to subjects with CAD, thus results cannot be extrapolated to non-CAD subjects. Measuring the levels of ADORA1, ADRA2A and PTGDS in CAD and non-CAD subjects would be of great interest to confirm their possible role in the particular characteristics of EAT and to improve our knowledge about the functions of this adipose tissue. Second, as we measured mRNA expression only, quantification of protein levels may reinforce or weaken the results observed, since protein levels can vary according to post-transcriptional and post-translational processes. Third, the sample size used in the microarray experiment is small. Four genes were robustly confirmed by qPCR in a larger sample size and similar experiments will be required to confirm all other genes claimed significant in the microarray data set. Finally, we did not take into account the various cell types which composed the different fat depots. Adipose tissue contains adipocytes, preadipocytes, fibroblasts, vascular cells, and macrophages. This heterogeneity may modify gene expression levels depending on the proportion of each cell type. In fact, our gene expression data seems to confirm previous observations showing a greater proportion of B and T cells in EAT and MAT compared to SAT (Figure S3) [10], [55]. Accordingly, genes differentially expressed in this study may reflect the different proportion of cell types in adipose tissue depots. Nonetheless, this study identified several genes with potential implications in the pathogenesis of heart disorders that are differentially regulated in EAT.

In conclusion, the gene expression profiles of EAT and MAT are more alike compared to SAT. An important proportion of genes involved in inflammation are differentially up-regulated in EAT and MAT compared to SAT, confirming that visceral adipose tissue may participate to heart diseases, including CAD. Our study also confirms the differentially up-regulation of PTGDS in EAT compared to MAT and SAT. To the best of our knowledge, it is the first time that the higher expression of ADORA1 and the lower expression of ADRA2A in EAT compared to SAT is documented, suggesting that the balance between these two genes may account for some of the regional differences observed in lipolysis. The differential up-regulation of PTGDS and ADORA1 suggests a possible cardioprotective role of EAT toward CAD, hypertension, and other cardiovascular dysfunctions. Moreover, the higher expression of genes involved in the arachidonic acid pathway in EAT may influence the pro/anti-inflammatory properties of this tissue and will require additional investigations. Overall, our results provide insights about the biology of EAT and its potential implication in CAD.

## Methods

### Ethics Statement

A written informed consent was obtained from all participants. The study was approved by the ethics committee of the Institut universitaire de cardiologie et de pneumologie de Québec.

### Study population

A total of 31 predominantly overweight men who underwent coronary artery bypass grafting (CABG) at the Institut universitaire de cardiologie et de pneumologie de Québec participated in the study. The subjects presented 1 to 3 affected vessels. The clinical characteristics of the subjects involved in the microarray and qPCR validation experiments are summarized in Table 1. The inclusion and exclusion criteria were the same for both groups. Subjects were consecutively selected first for the microarray experiment and then the qPCR experiment based on tissues availability. To reduce heterogeneity, only adipose tissues from white male subjects were selected. The average age of subjects in the microarray experiment was 66 years (range 48 to 76 years). All subjects had a body mass index (BMI)<40 kg/m^2^, were non-smokers or had stopped smoking for at least 6 months. The exclusion criteria were impaired renal function, chronic inflammatory or auto-immune diseases, aortic or mitral valve replacement, cancer, insulin treatment, and/or chronic obstructive pulmonary disease.

BMI was calculated as weight in kilograms divided by height in meters squared [56]. Obesity, overweight, and normal weight were defined as BMI≥30 kg/m^2^, 25 to 29.9 kg/m^2^, and 20 to 24.9 kg/m^2^, respectively. Waist circumference was obtained using a measuring tape directly on the skin with the subject standing. Measurements were taken at the end of expiration at the level midway between the lower rib margin and the iliac crest. The diagnosis of hypertension was based on a resting systolic or diastolic blood pressure >140 or >90 mmHg, respectively, or an actual hypertensive treatment. Dyslipidemia was defined as low-density lipoprotein cholesterol ≥3 mmol/L or the utilization of hypolipidemic agents. Nine out of 31 subjects had a clinical diagnosis of type 2 diabetes. Clinical characteristics of patients between the microarrays and the qPCR groups were tested using t-tests or Fisher's exact tests as appropriate. Pearson correlation tests were used to assess the relationship between gene expression and BMI.

### Adipose tissue collection

Adipose tissues from 3 compartments, namely epicardial, mediastinal, and subcutaneous, were taken from the chest of each individual. EAT correspond to the adipose depot in direct contact with the heart located between the myocardium and the visceral pericardium. MAT was defined as the fat within the mediastinum, outside the pericardial sac. The samples were collected from surgeries performed between October 2007 and August 2009. Immediately after resection, the tissues were snap-frozen in liquid nitrogen and stored in a local biobank at −80°C until RNA isolation.

### RNA extraction

RNA was isolated from 100 mg of tissue using the RNeasy Lipid Tissue Mini Kit (QIAGEN, Mississauga, Ontario) according to manufacturer's instructions. Before proceeding with microarrays experiments, the integrity of the RNA was assessed using the Agilent 2100 Bioanalyzer (Agilent technologies, Santa Clara, California) (Table S7). RNA concentration and purity for the quantitative real-time PCR (qPCR) validation was measured by the GeneQuant pro UV/Vis Spectrophotometer (Biochrom, Cambridge, UK).

### Microarrays

Adipose tissues of 6 subjects were chosen for whole-genome gene expression analysis. For each subject, gene expression was evaluated in the 3 adipose tissue compartments using the HumanWG-6 v3.0 expression BeadChips (Illumina, San Diego, California). The latter interrogated more than 48,000 probes derived from human genes in the NCBI RefSeq and UniGene databases. The RNA was labeled and hybridized using a standard Illumina protocol performed at the McGill University and Génome Québec Innovation Centre (http://www.gqinnovationcenter.com). Briefly, RNA extracted from the adipose tissue was converted to single-stranded cDNA using an oligo (dT) tagged with a phage T7 enabling the amplification and labeling of the cDNA by an in vitro transcription reaction which incorporates biotin UTP. The labeled cRNA was then hybridized to the chip at 58°C overnight, and washed the following day. Signal was developed using streptavidin-Cy3 and the chip was scanned by the Illumina BeadArray Reader. The GenomeStudio™ software was used to generate raw data resulting in standard file formats containing the signal values per probe. The complete data set has been deposited in the National Center for Biotechnology Information's Gene Expression Omnibus repository [57] and are accessible through GEO Series accession number GSE24425 (http://www.ncbi.nlm.nih.gov/geo/query/acc.cgi?acc=GSE24425).

### Microarray analysis

The raw data was log_2_-transformed and quantile normalized using the lumi package in R [58], [59]. All probes were considered in the analyses and no filter was applied to eliminate genes with low expression signals in one or many samples. The Significance Analysis of Microarrays (SAM) method [60] was utilized to identify probes differentially expressed among three pairwise comparisons (EAT vs MAT, EAT vs SAT, and MAT vs SAT). The false discovery rate (FDR) and the fold change threshold were set at 5% and 2.0, respectively. A false discovery rate of 10% was also explored for the comparison between EAT vs MAT. The fold change was obtained by raising 2 to the power of the mean difference in expression between any two adipose tissue compartments. Each probe was treated independently and transcripts interrogated by multiple probes were not summarized.

### qPCR

qPCR was used to validate the expression of four significant genes in EAT, SAT and MAT of 25 subjects (hence referred to as qPCR subjects) as well as the 6 microarrays subjects. The QuantiTect Reverse Transcription Kit (QIAGEN) was used to synthesize cDNA from 2 µg of RNA of each sample as described by the manufacturer. GAPDH was utilized as a reference gene as previously used in gene expression studies conducted in adipose tissues [10], [47], [61], [62], [63]. The latter gene was interrogated by three probes in the microarray experiment and similar signal intensities were observed in the three adipose tissues. The primers were designed using the software Primer3 v.0.4.0 (http://frodo.wi.mit.edu/primer3) and synthesized by Integrated DNA Technologies (Toronto, Ontario). PCR primers were tested In Silico using BLAT in UCSC to confirm their binding to a unique region of the genome and the absence of polymorphism. The genes, forward (F) and reverse (R) primer sequences used for qPCR were GAPDH (F: 5′- ATGTTCGTCATGGGTGTGAA and R: 5′-GGTGCTAAGCAGTTGGTGGT), ADORA1 (F: 5′-GCGAGTTCGAGAAGGTCATC and R: 5′-GCTGCTTGCGGATTAGGTAG), ADRA2A (F: 5′-CGACCAGAAGTGGTACGTCA and R: 5′-GTAGATGCGCACGTAGACCA), LIPE (F: 5′-GAGTTAAGTGGGCGCAAGTC and R: 5′-AAGTCCCTCAGGGTCAGGTT), and PTGDS (F: 5′-AACCAGTGTGAGACCCGAAC and R: 5′-AGCGCGTACTGGTCGTAGTC). The lengths of the amplicons were between 88 and 131 bp. The same cDNA sample was used to prepare the standard curves for each gene and was made from a pool of 36 SAT samples. For each gene, the experimental samples were tested in triplicate using the Rotor-Gene 6000 (Corbett Life Science, Concorde, Australia) in a final reaction volume of 20 µl containing 5 µl of 50× diluted cDNA, 10 µl of 2× QuantiTect SYBR Green PCR Kit (QIAGEN), and primers (0.3 µM for ADORA1, GAPDH, LIPE, and PTGDS; and 0.5 µM for ADRA2A). The final concentration of MgCl_2_ was 3 mM for ADORA1 and 2.5 mM for the other genes. The qPCR conditions were the same for all genes, 95°C for 15 min, and then 50 cycles at 94°C for 10 sec, 59°C for 30 sec, and 72°C for 30 sec. A melting curve analysis was performed at the end of each run and all showed a single peak, indicating specificity of the amplified products. According to the Rotor-gene 6000 series software v1.7 (Corbett Life Science), all standard curves had a correlation coefficient and efficiency higher than 0.98 and 0.9, respectively. For each gene, the nanograms of cDNA of each sample were calculated according to the standard curve method and normalized to GAPDH. The fold changes were obtained by dividing nanograms of cDNA between two groups of tissues (e.g. EAT vs SAT). Paired t-tests were used to assess significance differences in gene expression between any pairwise comparisons.

### Mapping of genes to biological pathways

The Ingenuity Pathway Analysis system (IPA) (Ingenuity Systems, www.ingenuity.com) was used to identify the principal biofunctions and diseases among the differentially expressed genes. Three input files were uploaded in IPA, one by pairwise comparison. The input files included the 48804 probes present in the microarrays, with their respective fold changes and the significant q-values obtained from SAM. Core analyses were then performed to overlay the results of each comparison into the Ingenuity Knowledge Base in order to identify particular diseases, molecular or cellular functions, metabolic and cell signaling pathways that were particularly enriched for genes claimed significant in our microarray experiment. In addition to IPA, the lists of significant genes were interpreted using the PANTHER classification system [64], [65] and the ToppFun tool in the ToppGene Suite [66].

## Supporting Information

Figure S1 A) EAT, B) MAT, and C) SAT intrapair correlation coefficients obtained from normalized expression data.
(PNG)

Figure S2 Venn diagrams showing the number of genes differentially expressed in the three pairwise comparisons. A) and B) shows the number of differentially up- and down-regulated genes, respectively. There was no significant gene down-regulated in EAT compared to MAT.
(JPG)

Figure S3 Heat map showing the expression of inflammatory and immune cell markers in the microarray experiment. A) EAT vs MAT, B) EAT vs SAT, and C) MAT vs SAT. The samples and genes are illustrated in columns and rows, respectively. Red and green represent high and low expression, respectively. Gene symbols are provided on the right side of each panel with cell type specificity in parentheses. The fold changes are indicated in parentheses. The asterisks represent probes that are claim significant based on the microarray experiment.
(TIF)

Table S1 SAM output listing all the significant up- and down-regulated probes in the comparisons EAT vs MAT.
(XLS)

Table S2 SAM output listing all the significant up- and down-regulated probes in the comparisons EAT vs SAT.
(XLS)

Table S3 SAM output listing all the significant up- and down-regulated probes in the comparisons MAT vs SAT.
(XLS)

Table S4 Top 10 genes significantly up- and down-regulated in EAT vs MAT.
(DOC)

Table S5 Top 10 genes significantly up- and down-regulated in EAT vs SAT.
(DOC)

Table S6 Top 10 genes significantly up- and down-regulated in MAT vs SAT.
(DOC)

Table S7 RNA extraction yields and RNA integrity of the microarrays samples measured with the 2100 Bioanalyzer (Agilent).
(DOC)
