# Supplementary figures and images for: The Transcriptome of Human Epicardial, Mediastinal and Subcutaneous Adipose Tissues in Men with Coronary Artery Disease
Source: PLoS One. 2011 May 16;6(5):e19908. doi: 10.1371/journal.pone.0019908 (PMC3095619; doi:10.1371/journal.pone.0019908)

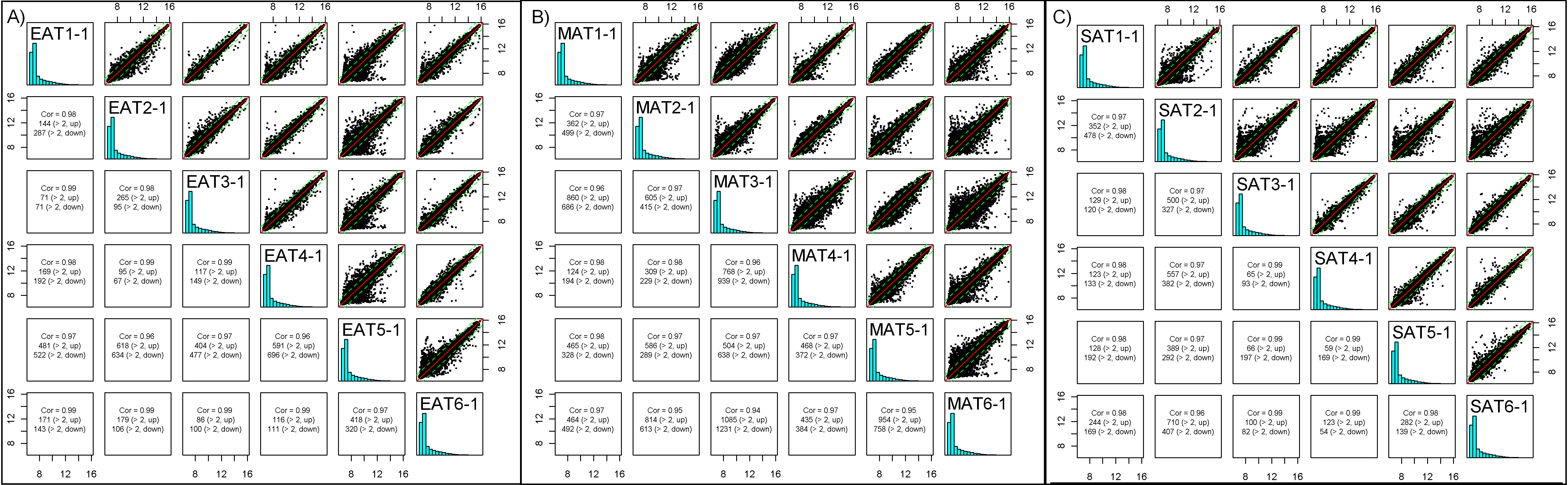

Supplement: Figure S1 — A) EAT, B) MAT, and C) SAT intrapair correlation coefficients obtained from normalized expression data. (PNG) [file pone.0019908.s001.png]

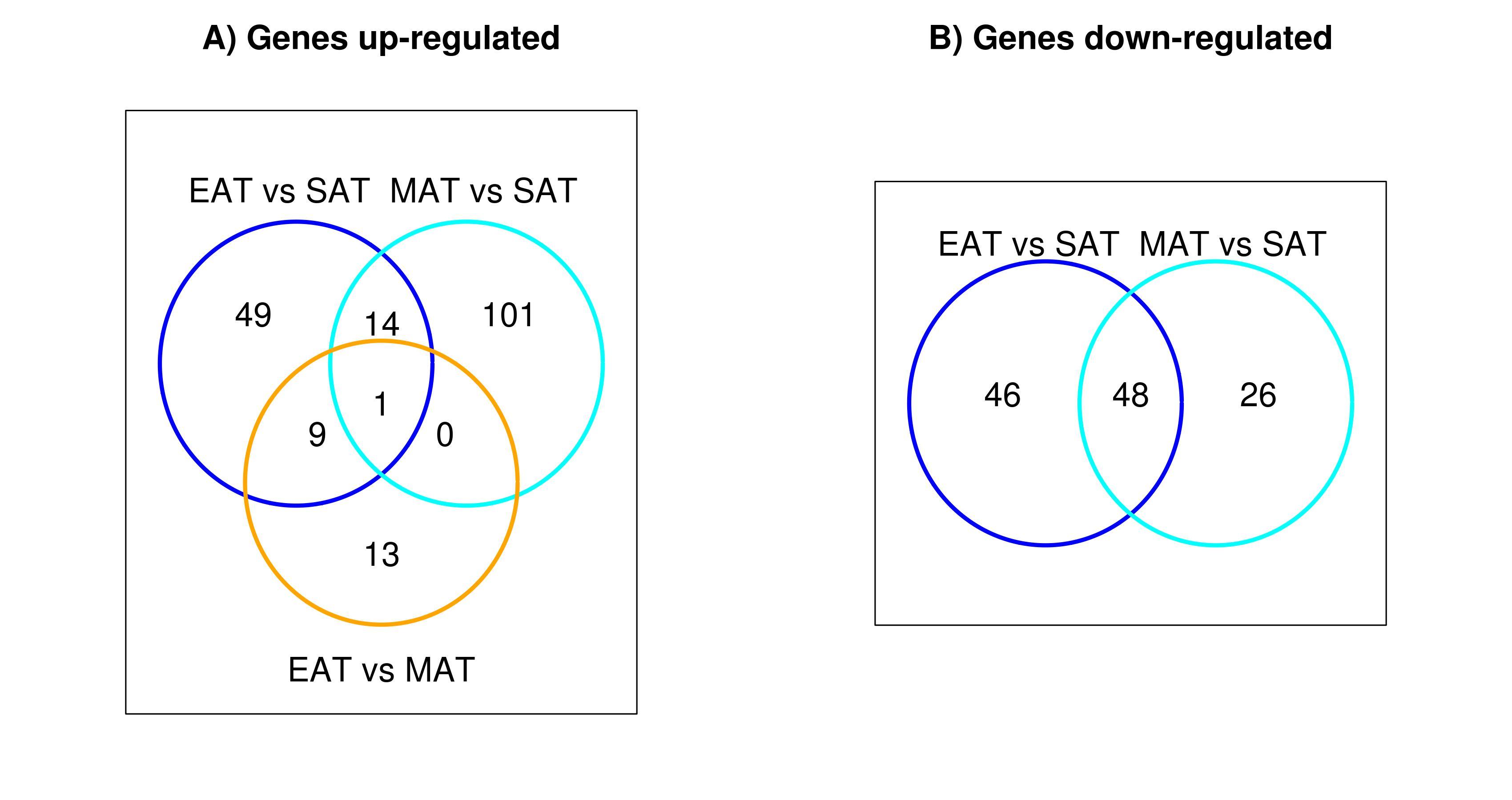

Supplement: Figure S2 — Venn diagrams showing the number of genes differentially expressed in the three pairwise comparisons. A) and B) shows the number of differentially up- and down-regulated genes, respectively. There was no significant gene down-regulated in EAT compared to MAT. (JPG) [file pone.0019908.s002.jpg]

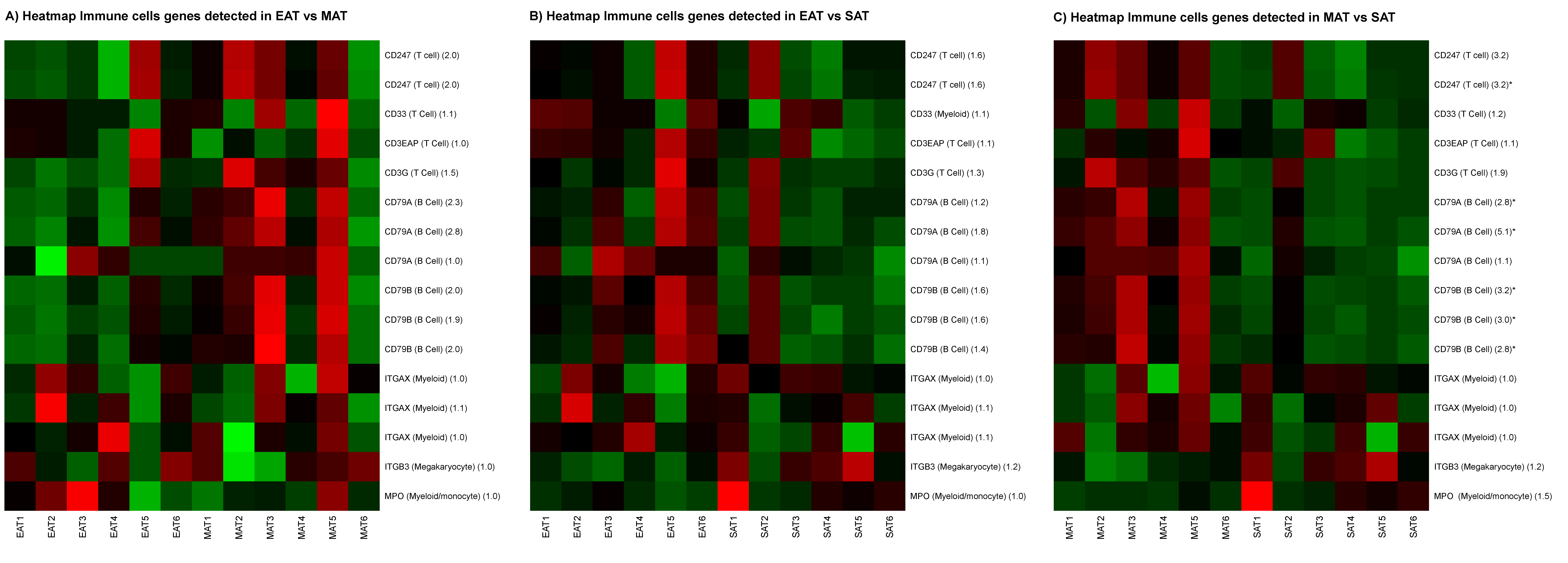

Supplement: Figure S3 — Heat map showing the expression of inflammatory and immune cell markers in the microarray experiment. A) EAT vs MAT, B) EAT vs SAT, and C) MAT vs SAT. The samples and genes are illustrated in columns and rows, respectively. Red and green represent high and low expression, respectively. Gene symbols are provided on the right side of each panel with cell type specificity in parentheses. The fold changes are indicated in parentheses. The asterisks represent probes that are claim significant based on the microarray experiment. (TIF) [file pone.0019908.s003.tif]
