# Supplementary material for: The Transcriptome of Human Epicardial, Mediastinal and Subcutaneous Adipose Tissues in Men with Coronary Artery Disease
Source: PLoS One. 2011 May 16;6(5):e19908. doi: 10.1371/journal.pone.0019908 (PMC3095619; doi:10.1371/journal.pone.0019908)
Supplement: Table S4 — Top 10 genes significantly up- and down-regulated in EAT vs MAT. (DOC) [file pone.0019908.s007.doc]

**Table S4**. Top 10 genes significantly differentially up- and down-regulated in EAT vs MAT.

| **Gene Symbol** | **Full Name** | **Biological Process** | **EAT-MAT** | **MAT-SAT** | **EAT-SAT** |
| --- | --- | --- | --- | --- | --- |
| **Differentially Up-regulated genes** | | | | | |
| TCF21 | Transcription factor 21 | mRNA transcription regulation | 4.04 | 1.55 | 6.28 |
| CDH19 | Cadherin 19, type 2 | Cell adhesion-mediated signaling | 3.30 | 1.25 | 4.13 |
| SERPINA5 | Serpin peptidase inhibitor, clade A (alpha-1 antiproteinase, antitrypsin), member 5 | Proteolysis | 3.30 | -1.89 | 1.74 |
| ADORA1 | Adenosine A1 receptor | G-protein mediated signaling; Macrophage and granulocyte-mediated immunity; Muscle contraction; Induction of apoptosis; Regulation of vasoconstriction, dilation | 2.92 | -1.19 | 2.45 |
| TFF3 | Trefoil factor 3 (intestinal) | Cell surface receptor mediated signal transduction; Cell motility; Defense response | 2.69 | 1.59 | 4.27 |
| TRIM55 | Tripartite motif-containing 55 | Proteolysis | 2.63 | 1.45 | 3.83 |
| KANK4 | KN motif and ankyrin repeat domains 4 | Biological process unclassified | 2.62 | -1.01 | 2.60 |
| G0S2 | G0/g1switch 2 | Cell cycle | 2.51 | -2.21 | 1.14 |
| MRAP | Melanocortin 2 receptor accessory protein | Positive regulation of cAMP biosynthetic process; Protein localization at cell surface | 2.33 | -1.65 | 1.41 |
| CGNL1 | Cingulin-like 1 | Muscle contraction | 2.32 | -1.01 | 2.30 |
| **Differentially Down-regulated genes*** | | | | | |
| ACTG2 | Actin, gamma 2, smooth muscle, enteric | Exocytosis; Endocytosis; Transport; Cytokinesis | -4.95 | -1.19 | -5.89 |
| HOXA5 | Homeobox A5 | mRNA transcription regulation; Segment specification | -4.04 | -1.95 | -7.89 |
| HOXC6 | Homeobox C6 | mRNA transcription regulation; Segment specification | -3.51 | -3.29 | -3.51 |
| CR2 | Complement component (3d/Epstein Barr virus) receptor 2 | Complement activation; Immune response | -3.41 | 4.35 | 1.28 |
| CETP | Cholesteryl ester transfer protein, plasma | Cholesterol metabolic process; Phospholipid homeostasis; Triglyceride homeostasis | -3.05 | 1.99 | -1.53 |
| VPREB3 | Pre-B lymphocyte 3 | Biological process unclassified | -3.00 | 3.92 | 1.31 |
| KIAA1199 | Kiaa1199 | Biological process unclassified | -2.99 | 5.41 | 1.81 |
| CD19 | CD19 molecule | B cell receptor signaling pathway; Cellular defense response | -2.99 | 4.64 | 1.55 |
| UBD | Ubiquitin D | Protein ubiquitination | -2.95 | 3.92 | 1.33 |
| BCL11A | B-cell CLL/lymphoma 11A (zinc finger protein) | B and T cell differentiation; Regulation of transcription | -2.85 | 4.01 | 1.41 |

*The top ten genes down-regulated in EAT vs MAT were not significant.
