# Supplementary material for: The Transcriptome of Human Epicardial, Mediastinal and Subcutaneous Adipose Tissues in Men with Coronary Artery Disease
Source: PLoS One. 2011 May 16;6(5):e19908. doi: 10.1371/journal.pone.0019908 (PMC3095619; doi:10.1371/journal.pone.0019908)
Supplement: Table S5 — Top 10 genes significantly up- and down-regulated in EAT vs SAT. (DOC) [file pone.0019908.s008.doc]

**Table S5.** Top 10 genes significantly differentially up- and down-regulated in EAT vs SAT.

| **Gene Symbol** | **Full name** | **Biological Process** | **EAT-SAT** | **EAT-MAT** | **MAT-SAT** |
| --- | --- | --- | --- | --- | --- |
| **Differentially Up-regulated genes** | | | | | |
| IGKV3D-20 | Immunoglobulin kappa variable 3D-20 | Immune response | 10.73 | -1.50 | 16.05 |
| IGLL1 | Immunoglobulin lambda-like polypeptide 1 | B-cell- and antibody-mediated immunity | 9.25 | -1.46 | 13.55 |
| IGJ | Immunoglobulin J polypeptide, linker protein for immunoglobulin alpha and mu polypeptides | Immune response | 7.66 | -1.66 | 12.69 |
| PTGDS | Prostaglandin D2 synthase 21kda (brain) | Fatty acid biosynthesis; Lipid metabolism; Intracellular signaling cascade; Transport; Muscle contraction | 7.65 | 2.01 | 3.81 |
| TCF21 | Transcription factor 21 | mRNA transcription regulation | 6.28 | 4.04 | 1.55 |
| CCL21 | Chemokine (C-C motif) ligand 21 | Cytokine/chemokine mediated immunity; Inflammatory response | 5.12 | 1.26 | 4.05 |
| RARRES1 | Retinoic acid receptor responder (tazarotene induced) 1 | Negative regulation of cell proliferation | 4.57 | 1.76 | 2.59 |
| TFF3 | Trefoil factor 3 (intestinal) | Cell surface receptor mediated signal transduction; Cell motility; Defense response | 4.27 | 2.69 | 1.59 |
| CDH19 | Cadherin 19, type 2 | Cell adhesion-mediated signaling | 4.13 | 3.30 | 1.25 |
| TRIM55 | Tripartite motif-containing 55 | Proteolysis | 3.83 | 2.63 | 1.45 |
| **Differentially Down-regulated genes** | | | | | |
| HOXB7 | Homeobox B7 | mRNA transcription regulation; Segment specification | -4.74 | -1.44 | -3.29 |
| DEFA1 | Defensin, alpha 1 | Chemotaxis; Immune response | -5.22 | 1.11 | -5.79 |
| CTHRC1 | Collagen triple helix repeat containing 1 | Complement-mediated immunity | -5.27 | -1.12 | -4.70 |
| ACTG2 | Actin, gamma 2, smooth muscle, enteric | Exocytosis; Endocytosis; Transport; Cytokinesis | -5.89 | -4.95 | -1.19 |
| HOXC8 | Homeobox C8 | mRNA transcription regulation | -6.69 | -1.67 | -4.02 |
| HOXA5 | Homeobox A5 | mRNA transcription regulation; Segment specification | -7.89 | -4.04 | -1.95 |
| NNAT | Neuronatin | Neurogenesis | -8.00 | -2.34 | -3.42 |
| CXCL14 | Chemokine (C-X-C motif) ligand 14 | Cell-cell signaling; Chemotaxis; Immune response | -8.10 | -2.45 | -3.31 |
| RN7SK | RNA, 7SK small nuclear | Biological process unclassified | -9.72 | -2.47 | -3.93 |
| HOXC6 | Homeobox C6 | mRNA transcription regulation; Segment specification | -11.57 | -3.51 | -3.29 |
