# Supplementary material for: The Transcriptome of Human Epicardial, Mediastinal and Subcutaneous Adipose Tissues in Men with Coronary Artery Disease
Source: PLoS One. 2011 May 16;6(5):e19908. doi: 10.1371/journal.pone.0019908 (PMC3095619; doi:10.1371/journal.pone.0019908)
Supplement: Table S6 — Top 10 genes significantly up- and down-regulated in MAT vs SAT. (DOC) [file pone.0019908.s009.doc]

**Table S6**. Top 10 genes significantly differentially up- and down-regulated in MAT vs SAT.

| **Gene Symbol** | **Full name** | **Biological Process** | **MAT-SAT** | **EAT-MAT** | **EAT-SAT** |
| --- | --- | --- | --- | --- | --- |
| **Differentially Up-regulated genes** | | | | | |
| IGKV3D-20 | Immunoglobulin kappa variable 3D-20 | Immune response | 16.05 | -1.50 | 10.73 |
| IGLL1 | Immunoglobulin lambda-like polypeptide 1 | B-cell and antibody-mediated immunity | 13.55 | -1.46 | 9.25 |
| IGJ | Immunoglobulin J polypeptide, linker protein for immunoglobulin alpha and mu polypeptides | Immune response | 12.69 | -1.66 | 7.66 |
| IGLL3 | Immunoglobulin lambda-like polypeptide 3 | Immune response | 5.88 | -2.72 | 2.16 |
| KIAA1199 | Kiaa1199 | Biological process unclassified | 5.41 | -2.99 | 1.81 |
| FCRLA | Fc receptor-like A | B-cell- and antibody-mediated immunity; Macrophage-mediated immunity; Natural killer cell mediated immunity | 5.40 | -2.76 | 1.95 |
| CD79A | CD79a molecule, immunoglobulin-associated alpha | B cell activation, differentiation and proliferation; B cell receptor signaling pathway | 5.12 | -2.80 | 1.83 |
| CD19 | CD19 molecule | B cell receptor signaling pathway; Cellular defense response | 4.64 | -2.99 | 1.55 |
| LTB | Lymphotoxin beta (TNF superfamily, member 3) | Cytokine/chemokine mediated immunity; T-cell, B-cell- and antibody-mediated immunity; Macrophage-mediated immunity; Natural killer cell mediated immunity; | 4.59 | -2.11 | 2.18 |
| CD3D | CD3d molecule, delta (CD3-TCR complex) | Cell surface receptor mediated signal transduction;Cell communication;T-cell mediated immunity | 4.56 | -2.18 | 2.09 |
| **Differentially Down-regulated genes** | | | | | |
| HBD | Hemoglobin, delta | Transport; Blood circulation and gas exchange | -3.33 | -1.38 | -4.59 |
| PPL | Periplakin | Keratinization | -3.41 | -1.05 | -3.57 |
| NNAT | Neuronatin | Neurogenesis | -3.42 | -2.34 | -8.00 |
| HOXB8 | Homeobox B8 | mRNA transcription regulation | -3.76 | -1.16 | -4.35 |
| RN7SK | RNA, 7SK small nuclear | Biological process unclassified | -3.93 | -2.47 | -9.72 |
| HOXC8 | Homeobox C8 | mRNA transcription regulation | -4.02 | -1.67 | -6.69 |
| EGFL6 | EGF-like-domain, multiple 6 | Cell adhesion and differentiation; Cell cycle | -4.06 | 1.34 | -3.02 |
| CTHRC1 | Collagen triple helix repeat containing 1 | Complement-mediated immunity | -4.70 | -1.12 | -5.27 |
| DEFA3 | Defensin, alpha 3, neutrophil-specific | Immune response | -4.73 | 1.03 | -4.59 |
| DEFA1 | Defensin, alpha 1 | Chemotaxis; Immune response | -5.79 | 1.11 | -5.22 |
