# Supplementary material for: The Transcriptome of Human Epicardial, Mediastinal and Subcutaneous Adipose Tissues in Men with Coronary Artery Disease
Source: PLoS One. 2011 May 16;6(5):e19908. doi: 10.1371/journal.pone.0019908 (PMC3095619; doi:10.1371/journal.pone.0019908)
Supplement: Table S7 — RNA extraction yields and RNA integrity of the microarrays samples measured with the 2100 Bioanalyzer (Agilent). (DOC) [file pone.0019908.s010.doc]

**Table S7.** RNA extraction yields and RNA integrity of the microarrays samples measured with the 2100 Bioanalyzer (Agilent).

| **Sample** | **Concentration (ng/ul)** | **rRNA Ratio (28s / 18s)** | **RIN** |
| --- | --- | --- | --- |
| SAT-1 | 91.4 | 1.4 | 7.7 |
| MAT-1 | 82.8 | 1.5 | 8.6 |
| EAT-1 | 164.8 | 1.3 | 8.4 |
| SAT-2 | 153.1 | 1.3 | 7.9 |
| MAT-2 | 232.0 | 1.5 | 9.1 |
| EAT-2 | 204.3 | 1.5 | 8.6 |
| SAT-3 | 54.8 | 1.6 | 8.7 |
| MAT-3 | 429.0 | 1.5 | 9.0 |
| EAT-3 | 165.3 | 1.4 | 8.5 |
| SAT-4 | 46.7 | 1.6 | 9.1 |
| MAT-4 | 116.0 | 1.6 | 8.8 |
| EAT-4 | 131.1 | 1.5 | 8.7 |
| SAT-5 | 89.7 | 1.4 | 8.5 |
| MAT-5 | 65.0 | 1.5 | 8.5 |
| EAT-5 | 275.6 | 1.4 | 9.0 |
| SAT-6 | 85.7 | 1.7 | 9.2 |
| MAT-6 | 218.8 | 1.4 | 8.7 |
| EAT-6 | 114.4 | 1.5 | 8.8 |

RIN: RNA integrity number
